# The effects of protected areas on the ecological niches of birds and mammals

**DOI:** 10.1038/s41598-022-15949-2

**Published:** 2022-07-08

**Authors:** Andrea Santangeli, Stefano Mammola, Aleksi Lehikoinen, Ari Rajasärkkä, Andreas Lindén, Marjo Saastamoinen

**Affiliations:** 1 Organismal and Evolutionary Biology Research Programme, Research Centre for Ecological Change, University of Helsinki, 00014 Helsinki, Finland; 2 LIBRe—Laboratory for Integrative Biodiversity Research, Finnish Museum of Natural History, University of Helsinki, Helsinki, Finland; 3 DarkMEG—Molecular Ecology Group, Water Research Institute, National Research Council of Italy (CNR), Verbania Pallanza, Italy; 4 The Helsinki Lab of Ornithology, Finnish Museum of Natural History, University of Helsinki, P.O. Box 17, 00014 Helsinki, Finland; 5 Metsähallitus, National Parks Finland, P.O. Box 81, 90101 Oulu, Finland; 6 Natural Resources Institute Finland (LUKE), P.O. Box 2, 00791 Helsinki, Finland; 7 Helsinki Institute for Life Sciences, University of Helsinki, 00014 Helsinki, Finland

**Keywords:** Biodiversity, Boreal ecology, Conservation biology, Ecological modelling

## Abstract

Protected areas are a cornerstone for biodiversity conservation, and typically support more natural and undisturbed habitats compared to unprotected lands. The effect of protected areas on intra-specific ecological niche has been rarely investigated. Here, we explore potential differences in ecological niche properties of birds and mammals across protected and unprotected areas, and relate such differences to species traits. We combine two decades of survey data of birds and mammals from protected and unprotected areas, and apply robust matching to obtain a set of environmentally comparable protected and unprotected sites. Next, we calculate intra-specific niche volume change and habitat shift between protected and unprotected areas, and use generalized linear mixed models to explain these responses with species traits (habitat specialization, body mass, diet, and red list status). The majority of bird and mammal species (83% and 90%, respectively) show different habitat use when occurring within and outside protected areas, with the magnitude of this shift highly varying across species. A minority of species (16% of birds and 10% of mammals) do not change their niche volume nor shift their habitat between protected and unprotected areas. Variation in niche properties is largely unrelated to species traits. Overall, the varying ecological niche responses of birds and mammals to protected areas underscore that there is no universal niche-based response, and that niche responses to land protection are species-specific.

## Introduction

The fields of conservation science and macroecology typically assume that all individuals of the same species should show similar ecological responses to environmental cues^1^—a pattern often referred to as niche conservatism^2,3^. However, individual heterogeneity, beyond the simple age and sex class differentiation, represent key components of ecological theory^4^. For example, individuals may differ in their morphological traits (e.g., body size^5^), in their behavioral responses to abiotic (e.g., environmental gradients) and biotic (e.g., competition, predation) factors, as well as in their use of resources^1^. These responses may, in turn, trigger variation in population demography^6,7^ and in the ecological niche of different populations^8^. A recent study unveiled a large variation in the environmental niche responses among conspecific individuals, and also that such responses are structured within populations^9^. Such examples underscore the importance of studying ecological niche responses at the intra-specific level. In this study, the ecological niche is intended as the *n*-dimensional hypervolume, or an abstract Euclidean space determined by a set of independent axes which correspond to, for example, the environmental factors that affect the occurrence and fitness of an organism^10^.

Habitat loss, fragmentation and degradation are rampant across most unprotected land in the world, particularly in largely human-managed and densely populated regions^11^. Conversely, protected areas typically support more natural landscapes^12^, although management effectiveness plays a crucial role in preserving ecosystem quality within protected areas^13^. However, generally protected areas may offer more high quality resources to species inhabiting them. Therefore, we may expect that at least for some species the ecological niche would differ between protected and unprotected environments^1^. This difference may also be mediated by species traits^4^, like body mass, habitat or diet specialization^14^ and potentially the conservation status of the species^15^.

The role of protected areas has been largely quantified with focus on preserving ecosystem integrity and biodiversity^12,16^. Conversely, the ecological impact of protected areas on wildlife, with regards to their effects on the ecological niche of species, has been rarely studied. This research gap is particularly pertinent given the low representation of vertebrate species niche by the global protected area network^17^, and the strong ecological contrast between protected and unprotected lands worldwide^18^.

The Hutchinson’s *n*-dimensional hypervolume concept^10^ assumes that the niche encloses the range of conditions allowing a species to survive and reproduce. This concept has emerged as a simple and direct way to interpret the niche as a geometric shape^19^. The *n*-dimensional hypervolume concept has been applied to study different properties of the niche at various levels, from individuals, to species, to communities and ecosystems^19^. Recent advances in ecological niche modeling have made it possible to efficiently quantify and compare the niche of multiple species based on the calculation of the intersection between hypervolumes using several distance metrics and niche similarity indexes^20,21^. Specifically, a framework has been recently proposed to decompose the difference among hypervolumes into two elements: ‘Niche shift’, the replacement of space between different hypervolumes and ‘Niche contraction/expansion’, the differences in the amount of space occupied by different hypervolumes^22^. This approach has been successfully employed to disentangle the processes underlying niche differentiation in organisms as diverse as birds^22^, spiders^23^, pipefish^24^, rotifers^25^, and seagrasses^26^.

In this study, we investigate potential differences (or implicitly changes) in the ecological niche properties of birds and mammals across protected and unprotected areas, taking advantage of systematic wildlife monitoring data collected over two decades across Finland. We first match comparable protected and unprotected sites^27,28^, and we then partition the overall niche differentiation into the distinct components mentioned above, i.e. niche shift and niche contraction/expansion^22^. This allows to compare within-species niche differences between protected and unprotected areas. Specifically, we first quantify and compare the within-species niche expansion and habitat shift in protected and unprotected areas. Second, we assess whether the above measures of niche expansion and habitat shift are related to species traits, such as body mass, habitat and diet specialization, the proportion of vertebrates in the diet (a proxy for guild), as well as the conservation status.

**Figure 1:**
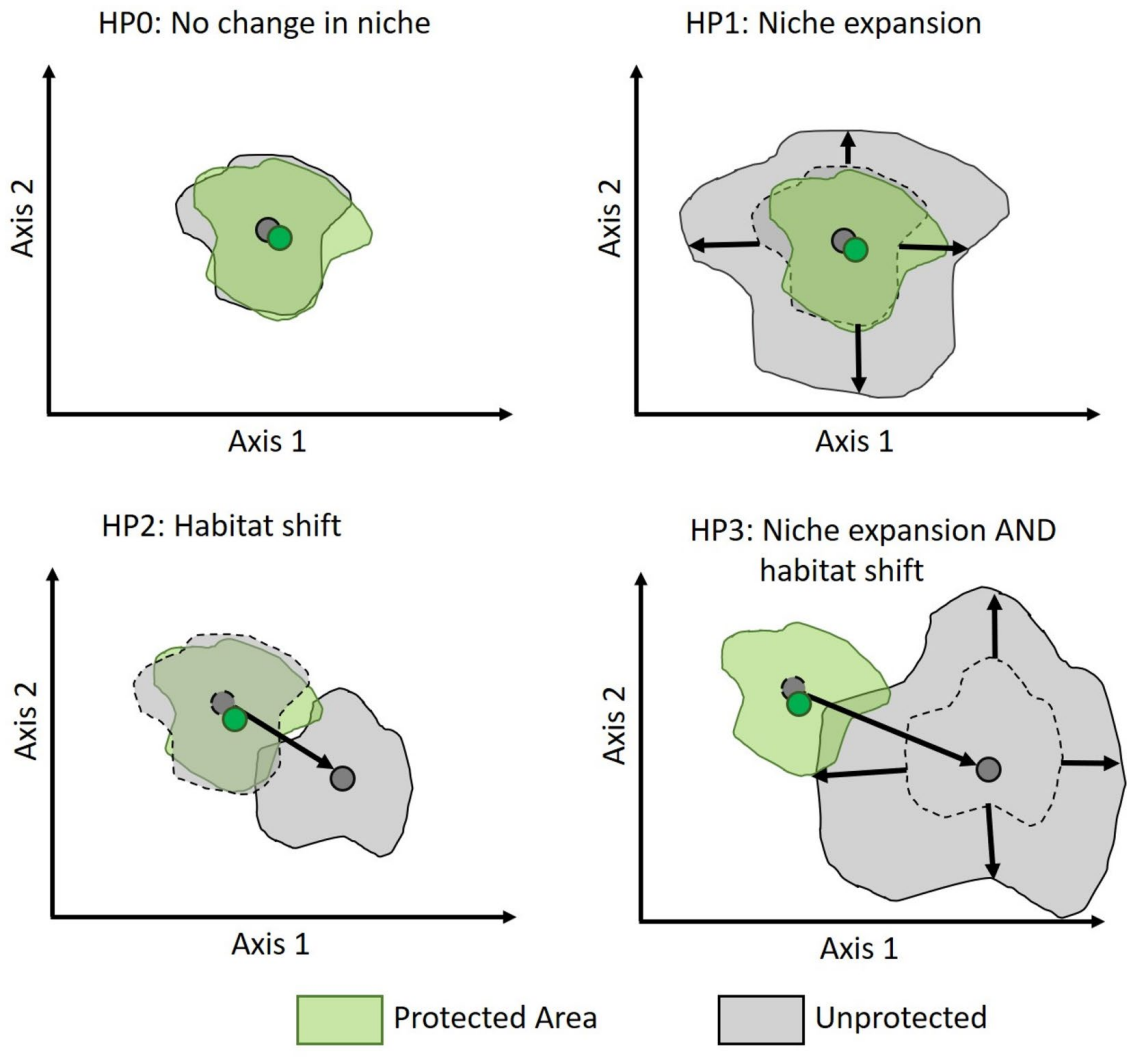
Schematic representation of our alternative hypotheses design. The null hypothesis (HP0) represents no change (difference) in the ecological niche between protected and unprotected areas within a certain species. Hypothesis 1 (HP1) represents a scenario of niche expansion outside protected areas (technically, this hypothesis also allows for niche contraction, although we do not expect it). Hypothesis 2 (HP2) represents a scenario where species shift their habitat between protected and unprotected areas. Hypothesis 3 (HP3) represents a scenario where species both expand (or contract) their niche volume outside protected areas and shift their habitat between protected and unprotected areas.

Within protected areas, species may more easily fulfill their ecological requirements within one or few optimal habitats as compared to more disturbed habitats outside of protected areas. As a result, we expect that the ecological niche of species will differ between protected and unprotected areas by two distinct mechanisms, or their combination (Fig. 1). Compared to protected areas, in unprotected land we can either observe an expansion of the niche volume size (hypothesis 1), a shift to use different habitats (hypothesis 2), or both (hypothesis 3). The null hypothesis is that there is no observable niche volume expansion nor niche shift between protected and unprotected areas (hypothesis 0). With regards to hypothesis 1, while we expect expansion of the niche volume from protected to unprotected areas, we technically allow for both expansion and contraction (see “Methods” section for more details). Moreover, we predict that the above patterns are linked to species traits based on the rationale as follows. Larger animals, animals at the top of the food chain (i.e. those with a higher proportion of the diet based on vertebrates) typically have higher ecological requirements, e.g. in terms of food, space or others^29^. In addition, generalist and non-threatened species may possess higher behavioral plasticity. All these species traits may thus mediate the differences in ecological niche properties between protected and unprotected areas. With regards to the conservation status of the species, we predict that threatened species, which are typically more reliant on protected areas and the ecosystems therein, may show smaller changes in the niche properties at the interface between protected and unprotected areas. Conversely, non-threatened species may be more plastic and adjust their niche properties according to the different environmental conditions present inside and outside of protected areas.

## Results

**Figure 2:**
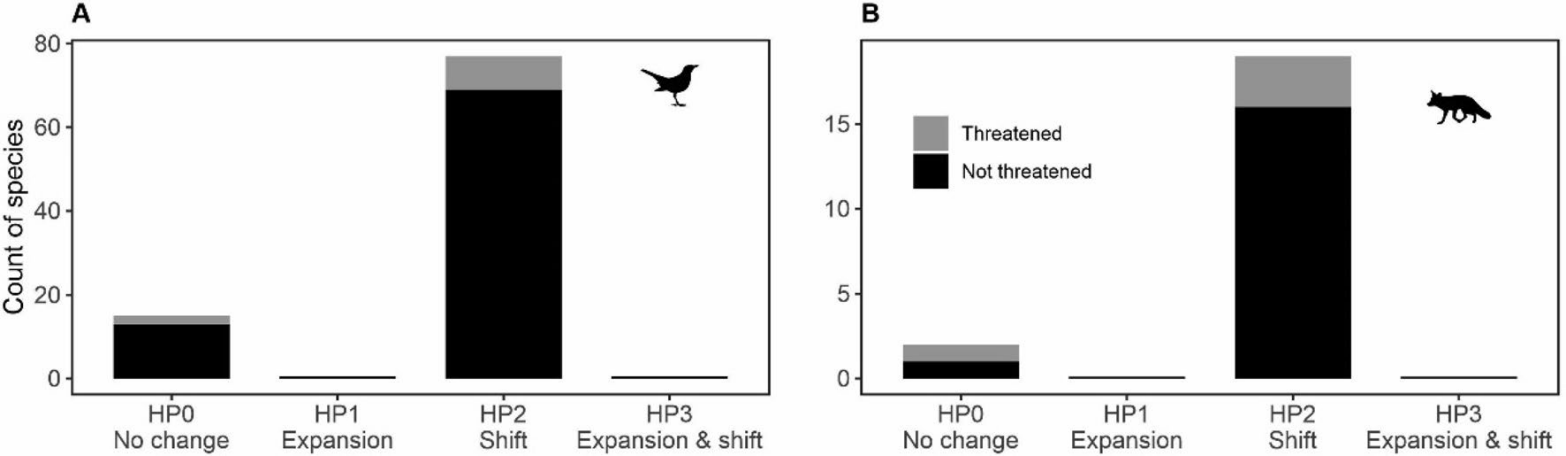
The number of bird (**A**) and mammal (**B**) species that fit each of the hypotheses as presented in Fig. 1. HP0 represents no expansion in the volume of the niche and no shift in habitat, HP1 represents only expansion in the volume, HP2 only shift in habitat, and HP3 represents both expansion in the volume of the niche and shift in habitat. A threshold of 0.20 (20%) was used to separate the continuous differentiation in the ecological niche, either volume or habitat shift, so that values < 0.20 represent no change for the specific niche metric, and values > 0.20 represent change. The number of threatened and non-threatened species is shown with different colors, and both sum to the total count of each bar. No species fitted HP1 nor HP3 in neither birds nor mammals.

**Figure 3:**
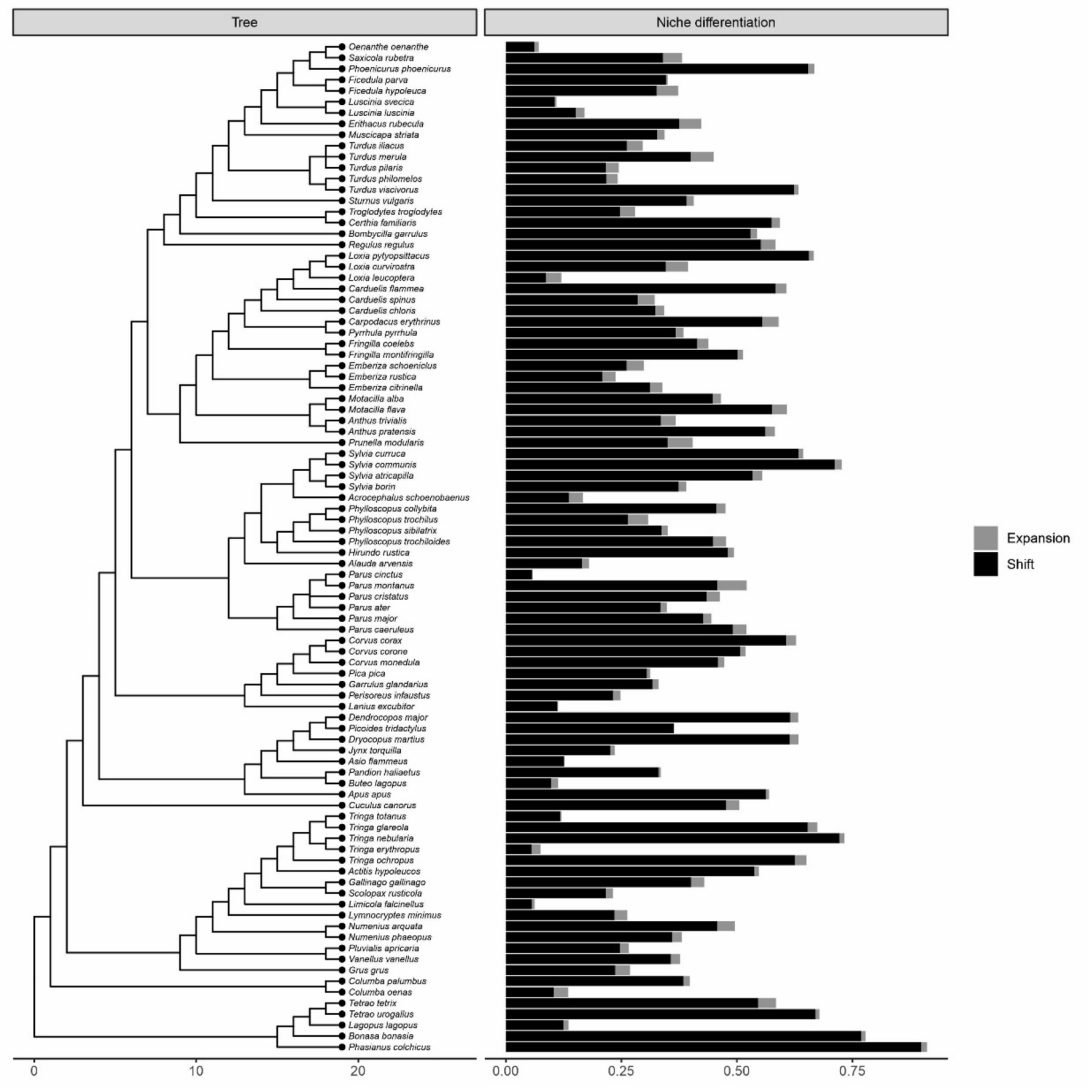
Species specific responses of birds in terms of habitat shift (black) and niche expansion (or contraction; grey). The sum of niche expansion / contraction and shift (depicted with different colors) gives the total niche differentiation between protected and unprotected areas, following Ref.^22^. The phylogeny of the species is shown in the left.

**Figure 4:**
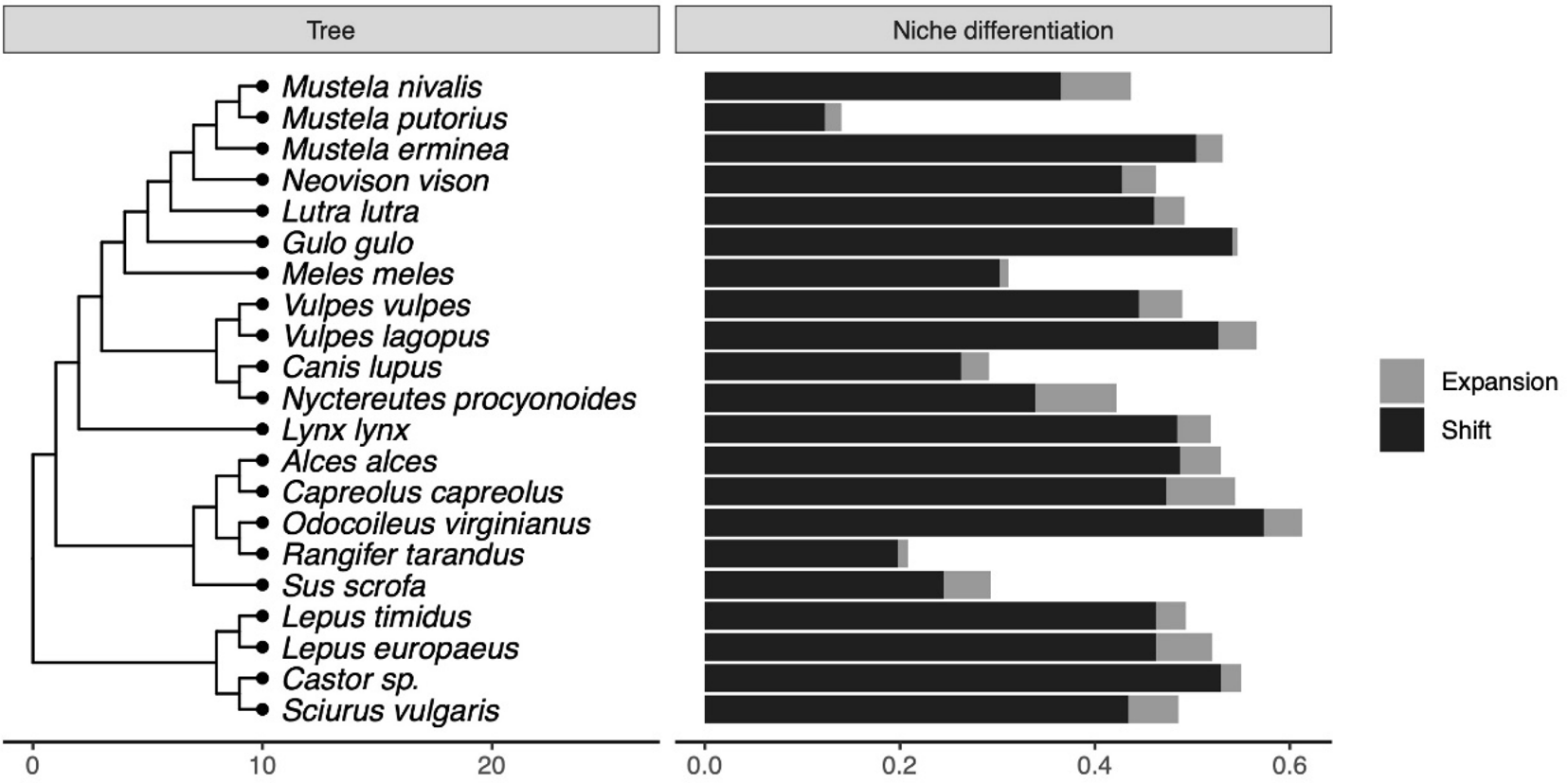
Species specific responses of mammals in terms of habitat shift (black) and niche expansion (or contraction; grey). The sum of niche expansion/contraction and shift (depicted with different colors) gives the total niche differentiation between protected and unprotected areas, following Ref.^22^. The phylogeny of the species is shown in the left.

The intraspecific comparison of the ecological niche characteristics within and outside protected areas revealed consistent patterns of change for both birds and mammals with regards to our initial hypotheses (Fig. 2). Specifically, when applying the threshold of 0.20 (20%) to separate the continuous differentiation in the ecological niche (i.e., values above 0.20 are considered change, and below 0.20 considered as no change), the hypothesis that was supported by the highest number of species was HP2, whereby a species shifts its habitat use between protected and unprotected sites. Namely, 77 species of birds (83%, out of the total of 93 species) and 19 species of mammals (90%, out of the total of 21 species) showed a measurable habitat shift supporting HP2. A remaining smaller number of 15 bird and 2 mammal species (16% and 10% of the total, respectively) showed no change in the ecological niche between protected and unprotected areas with respect to niche volume expansion and habitat shift, thereby complying with the null hypothesis HP0. No species of birds nor mammals conformed to HP1 or HP3, i.e., there was no support for niche volume expansion alone (HP1) nor in combination with niche shift (HP3). The above results are broadly robust to changes in the threshold used to separate change from no change (Support Fig. S5). High variation in the magnitude of habitat shifts within and outside protected areas was apparent for both birds (Fig. 3) and mammals (Fig. 4) species.

**Figure 5:**
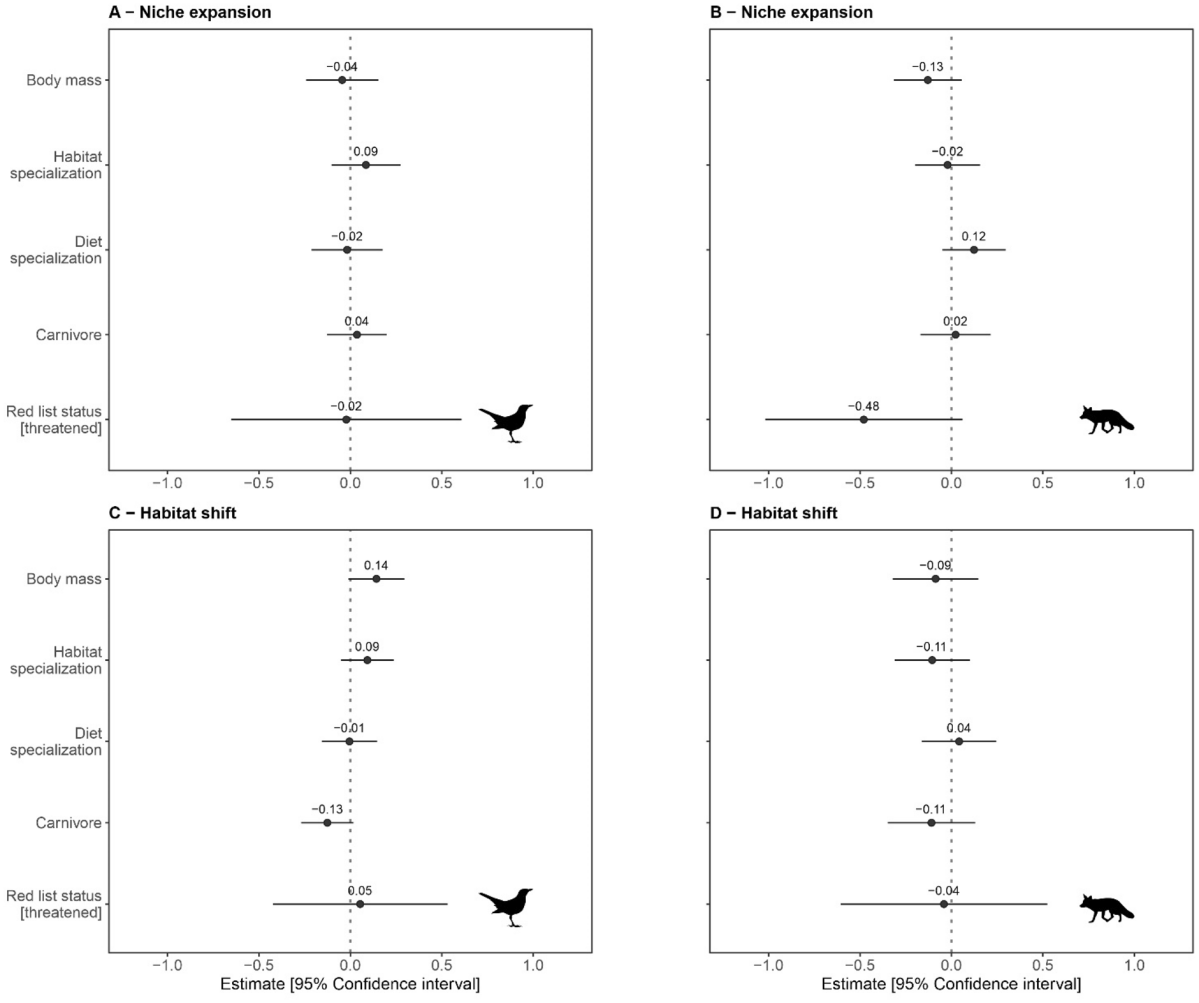
Relationships between species traits and ecological niche differentiation processes between protected and unprotected areas. (**A,B**) Represent niche expansion for birds and mammals, whereas (**C,D**) Represent habitat shift for birds and mammals, respectively. Each panel shows the estimated standardized coefficients β ± 95% CI, illustrating the effect of each trait. All continuous variables have been scaled to zero mean and 1 SD. For the red list status, the non-threatened status is used as a reference category. Carnivore indicates the proportion of the diet based on vertebrates (including endotherms and ectotherms). See Table S2 for the full test statistics results.

The analysis linking niche volume expansion and habitat shift to species traits revealed that niche volume and habitat shift are broadly unrelated to the species ecological traits considered here (Fig. 5, Table S2).

However, we found a weak correlation (p = 0.067) between the extent of niche shift and body mass in birds, with larger species showing marginally more habitat shifts (Fig. 5). Similarly, we found a weak indication (p = 0.084) that birds with a higher proportion of their diet based on vertebrates (largely raptors) exhibit a lower habitat shift when transitioning from protected to unprotected areas. Another weak signal (p = 0.081) suggests that red listed mammals change their niche volume less than non-threatened mammals at the interface between protected and unprotected areas.

Niche response was not phylogenetically structured (all Pagel’s λ < 0.05), indicating no unexplained phylogenetic structure in the residuals of the models.

## Discussion

Here we quantify and compare the within-species niche change (difference) between protected and unprotected areas, and relate such change to species traits. Among the three hypotheses tested (Fig. 1), we found strong support only for the habitat shift hypothesis (HP2), with the majority of species of birds and mammals showing different habitat use when living in protected compared to unprotected areas. This pattern was underscored by large interspecific variation in the magnitude of the shift. For a small number of species we found no change (difference) in the ecological niche between protected and unprotected areas (Fig. 2), emphasizing that there is not a universal niche-based response to the level of protection. Moreover, such responses, both in terms of habitat shift and niche volume, were largely unrelated to species ecological traits tested, namely habitat specialization, body mass, diet, and red list status.

Our study has some limitations that should be borne in mind when interpreting the results and drawing conclusions. On the methodological side, it is not possible, given the current analytical tools, to precisely identify the causes underlying the observed patterns of niche differentiation^22^. With respect to niche shift, using the currently available tools it is not possible to pinpoint the direction of the shift and it is difficult to identify the specific habitat axes that are causing the observed shifts at the species level. Furthermore, the 20% threshold used to separate niche change from niche stasis (Fig. 2) can be regarded as arbitrary, because niche change and niche stasis align along a gradient^30^. We however show that the overall results are rather robust to changes in the threshold level used to discriminate change from no change (see Support Fig. S5). Moreover, it is known that the resolution of the underlying data used to infer the niche may affect the detectability of niche changes, with finer resolutions typically enhancing detection of change^30^. In our case, having higher resolution in the wildlife survey data, e.g. using point counts rather than transects, could unveil patterns that may be hidden at the current resolution of analyses, and help identify the mechanisms driving the observed changes. Nevertheless, we contend the results are still instrumental in highlighting broad patterns of shift in the ecological niche of species between protected and unprotected areas.

### Niche responses to land protection are species-specific

Our analyses suggest that intraspecific niche differentiation between protected and unprotected areas occurred mostly by processes of niche shift rather than by niche volume contraction or expansion. Our design explicitly, and strictly, matched each protected site with a corresponding and comparable unprotected site based on similar amounts of each relevant habitat type, among all the matching criteria. Moreover, sites were also matched so that a protected site would only be paired to a correspondent unprotected site within the same vegetation zone, thereby minimizing biogeographical differences (e.g. due to a large latitudinal gradient). Bird survey sites in protected areas have also been matched to their unprotected counterparts based on similar sampling effort. Consequently, the observed shift cannot be attributed to methodological, biogeographical or habitat composition differences between protected and unprotected sites, because these have been largely minimized at the design (matching) stage. However, we did not match sites according to habitat quality (e.g., forest age, ecosystem intactness, which in turn may affect carrying capacity and resource availability) and configuration. Therefore, the observed patterns of niche shift between protected and unprotected areas may be, at least partly, attributed to mechanisms related to habitat quality, rather than quantity. For example, while coniferous forest types may be found in the same amount within and outside of protected areas (as a result of our matched design), species may find old-growth coniferous forests mostly within protected areas in Finland, whereas in unprotected land such forests are much younger (thus of lower ecological “quality”) due to commercial forestry. This may force the species to occupy different habitats outside of protected areas, such as mixed or deciduous forests. This is for example the case of the White-tailed sea eagle [*Haliaeetus albicilla* (L.)], which inside protected areas typically breeds in old growth coniferous forests supporting large trees, but outside of protected areas can use a range of conditions, including single standing trees in the middle of clear-cuts, or even nest on the ground on rocky islets^31^.

Rapid changes in the ecological niche in response to environmental factors, such as habitat quality, have been documented in a variety of species and habitats^30^, including animals and plants. Such findings largely question the assumption of niche conservatism, whereby species should preserve their niche across space and time^3^. However, many of the documented cases of niche shift involve species of plants and animals colonizing new geographical areas^32–35^. For example, niche shift is often a key factor enabling successful invasions by alien species^36^, although the opposite (i.e. niche conservatism) may also occur, especially for the most invasive species^37^.

### Niche responses to land protection are largely unrelated to traits

Our trait based analysis revealed no significant effect of any of the considered traits on the habitat shift and volume change for either birds or mammals. However, some weak relationships found may be worth discussing. In particular, among the birds, larger species exhibited marginally higher habitat shift from protected to unprotected areas, as compared to smaller species. Larger species typically require more resources^29^. On unprotected lands, larger bird species may be able to persist by shifting to different habitats in order to fulfill their life-cycle requirements, thereby contributing to the observed weak pattern. We also found that birds whose diet is largely composed of vertebrates (such as raptors and other scavengers) exhibit a marginally, but again not significantly, lower shift in habitat compared to birds whose diet largely lacks vertebrates (e.g. granivorous or insectivorous species). This result may relate to the fact that predators may seek to occupy the same preferred habitat, irrespective of the protection level, because this habitat supports their preferred prey^38^. Among the mammals, we found that threatened species exhibit less change in niche volume between protected and unprotected areas compared to non-threatened species. While any discussion on this finding is highly speculative, this result may indicate that threatened mammals are rather conservative in maintaining the same niche volume irrespective of the protection status of the land where they are found.

Ultimately, it is often challenging to predict the link between species traits and niche properties^39^, and this study confirms such challenges. For example, few studies have shown that species traits may not represent robust predictors of species responses to environmental change, and that site- and species-specific responses are challenging the identification of broad patterns at the large scale^40,41^.

### Conclusions

The representation of species niche by protected areas is gaining momentum^17^. The vast majority of vertebrate species have their ecological niche poorly represented within the global protected area network^17^. While this calls for a reconsideration of the criteria used for identifying protected areas, especially in light of the post-2020 Aichi Targets, there is also a strong need to quantify the effects of protected areas on species ecological niches. Such research, as the one presented here, could highlight patterns of species ecological niche shift that could inform conservation. For example, identifying the process, either niche expansion or habitat shift, driving niche change of each species, as well as the intrinsic and extrinsic traits mediating such changes, may help understand and predict species vulnerability to anthropogenic pressures^29^.

This study is among the first attempts to quantify how differences in species ecological niche in relation to protected areas. As the field of ecological niche modeling is rapidly expanding, with more tools becoming available, there will be more and more opportunities to test hypotheses on how protected areas contribute to shape, and therein to support, the ecological niche of species. Specifically, finding ways to deeply understand the direction of the shifts and pinpoint which habitat variables mostly contribute to the observed shifts will represent a critical step forward. Such improvements would ultimately allow more targeted conservation of key properties of species niche and enhance the long-term persistence of biodiversity.

## Methods

**Figure 6:**
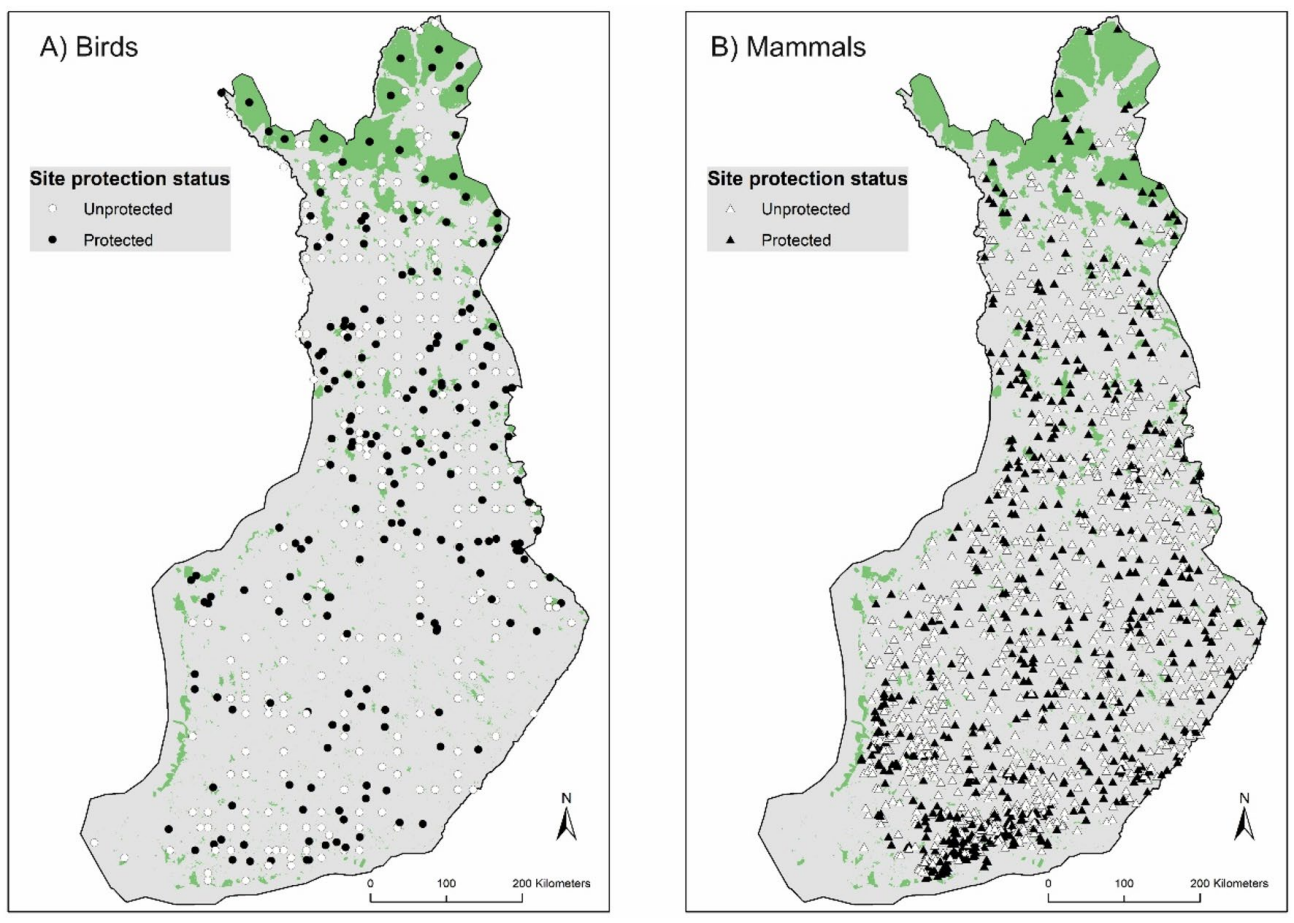
Distribution of the matched protected and unprotected sites for birds (left panel) and mammals (right panel) within Finland, with reference to the protected area network (areas shown in green color) and to the four main vegetation zones: Hemi Boreal (HB), South Boreal (SB), Middle Boreal (MB) and North Boreal (NB). These vegetation zones where used for matching protected sites to similar sites within the same vegetation zone (see “Methods” for more details). Note that many small protected areas are not well visible at this scale. For this reason, some protected sites may appear outside of the protected area network. Maps produced using ArcGIS 10.1 software (©ESRI). The protected area boundaries shown are derived from the open access resource at www.protectedplanet.net.

We restricted the study to the 21-year period 1999–2019. Through this period, we achieved the best coverage of bird and mammal data across Finland, both within and outside protected areas. Also, this is the only period when standardized and temporally comparable environmental data (i.e., land use across different habitat types, including forest and open landscapes) were available throughout the country (see below). Finland extends across over 1100 km in latitude and over 500 km in longitude. Land cover is largely dominated by forests (~ 78%), mostly coniferous forests, which are typically under intensive commercial forestry management regimes^42^. Agricultural areas cover ~ 7–9% of the country^43^, and are largely dominated by cereal cultivation (over 50% of the utilized agricultural area), fodder grassland (~ 25%), and scarce pastures (~ 3%). Lakes cover ~ 10% of the country. Protected areas are few but very large in the north of Finland, and small and numerous in the south (Fig. 6); they largely target the protection of old-growth forests and mires^44^.

### Bird survey data

We used a dataset consisting of bird observations recorded across Finland using the line transect method. The line transect counts—ongoing since the early 1970s^45^—include one visit, typically during the hours 3–10 a.m. from late May to early July, whereby all land-birds (including passerines and other major bird groups, such as raptors, woodpeckers, pigeons, grouse and so on) are counted along predetermined routes of 3–6 km each. The census unit is pairs, so e.g., a singing male, single female, a nest or a brood is transformed into one pair (see details in Refs.^46,47^). This method is appropriate for monitoring bird populations over large areas^46^. The bird survey data from unprotected land were available at the resolution of the single line transect per year. The data from protected areas were instead available as pooled observations from the transects within a given protected area in a given year [i.e., the number of pairs for each combination of bird species, unique protected area (site) and year]. The spatial sample unit in this case was the single protected area and not the line transect^44^. Consequently, the variation in overall length of line transects was relatively high for the protected area data as compared to unprotected area data. We explicitly account for this potential confounding factor at the design phase of the study (see “Matching protected and unprotected sites” section). Overall, the bird survey data from protected and unprotected areas are collected with the same standardized methodology and are fully comparable, as shown by recent work using the same data^47–49^.

### Mammal survey data

Similarly to the birds, the mammal data analyzed are from a national long-term population monitoring program, namely the Finnish Wildlife Triangle Scheme^50^. The scheme is based on permanent 12 km long line transect routes shaped as equilateral triangles (with 3 × 4 km sides). Each triangle is surveyed in winter (mid-January–mid-March) by local hunters who identify and count all fresh snow tracks crossing the transect line^50^. All game mammal species’ tracks are counted, including primarily ungulates, carnivores, hares, and squirrels (excluding e.g., small rodents, insectivores and bats). The number of snow tracks are related to transect length and number of snow days, during which the tracks have accumulated, i.e., days since last snowfall or days since the pre-count (where old tracks were mapped or marked to avoid recounting). The unit of the abundance index is No. tracks/10 km/snow day.

### Environmental variables and protected areas

To extract environmental variables around each bird and mammal survey location, we used the Corine Land Cover dataset (© European Union, Copernicus Land Monitoring Service, European Environment Agency EEA). We used all available Corine Land Cover years (2000, 2006, 2012, 2018), and associated each of these years with the wildlife survey data (see details below) obtained during the same year, as well as the year before and after each Corine Land Cover year. This resulted in four periods (1999–2001, 2005–2007, 2011–2013, 2017–2019). We re-classified the Corine Land Cover categories to obtain seven rather broad habitat classes that would be generally relevant to most of the species studied (see Support Table S1). Namely, artificial surfaces, agricultural land, broad leaved forest, coniferous forest, mixed forest, shrub and herbaceous vegetation, water (including sea and inland wetlands, covering also mires and bogs). Other land use classes were considered too rare and localized to be meaningfully included in this study, and we wanted to limit the number of variables as this is advised for multidimensional hypervolume analyses (the “curse of dimensionality” problem discussed in Ref.^51^). Because of this dimensionality problem, and also because we are interested in comparing the intra-specific niche within and outside of PAs, we ignored environmental variables other than land use. Such variables (e.g. climatic conditions) are likely spatially autocorrelated at scale of this study. This is because we matched protected with unprotected sites within the same vegetation zone, which also captures rather homogeneous bioclimatic conditions. Therefore, including these other dimensions would represent a very high cost in terms of niche model stability and result reliability, compared to likely marginal gain given the focus of this study. We derived the proportion of the coverage of each of the above seven land use classes within a 500 m buffer area around each bird line transect outside protected areas. Because the bird line transects inside protected areas were available only as pooled observations for each protected area and year, we used a circular buffer centered at the centroid point of each protected area to derive the proportion coverage of the seven land use classes. The radius of this circular buffer was 1331 m, which represents an area equal to the average area covered by buffers around unprotected surveys. While this area may be larger than that of the small protected areas considered in this study, such larger area may be still ecologically influenced by the presence of the protected areas via spillover mechanisms^52^. We however also extracted the cover of each of the seven habitat classes from across the entire area of the protected area. The correlation between the habitat cover as extracted using the radius, or using the entire protected area, was high for most of the variables (see Supplementary Fig. S1), indicating that this choice would have little influence on the final results. Based on these correlation results, and on the rationale given above, we therefore opted to use the habitat variables as extracted using the radius approach for bird surveys inside protected areas, because this would enhance the consistency and comparability in the area amount and its shape between protected and unprotected sites for the birds. For the mammal surveys, we used a buffer centered at the centroid of the triangle survey and with radius of 2809 m, which depicts the area of the circle crossing the three vertices of the triangle and an extended 500 m beyond that. The habitat surrounding the transect is hence measured at different scales for birds and mammals, which however, does not undermine the analysis of differences in ecological niche between and within species belonging to either taxonomic group (birds or mammals).

Moreover, each survey route was also assigned a status whether it was protected or unprotected. For the bird data, this information was already available because these data have been collated from two separate databases, one specific for the surveys inside protected areas, and one for those outside. Despite the above, the survey technique and protocol for bird observations were identical inside and outside protected areas. For mammals, we spatially intersected a circular buffer centered at the centroid of the triangle survey and with radius of 2309 m (which depicts the area of the circle crossing the three vertices) with a spatial layer of the protected areas (obtained from www.protectedplanet.org; version as of January 2021). Similar to Ref.^28^ we assigned each mammal survey route whose buffer intersected any protected area as protected, and the rest as unprotected. The initial number of unique line transects per period (that is, four 3-year periods on which following analyses are based, described above) was 1195 in unprotected land and 510 in protected areas for the birds, and 3141 in unprotected land and 1553 in protected areas for the mammals.

### Species traits

We classified each species as threatened (lumping together the IUCN categories Vulnerable, Endangered and Critically Endangered), or not threatened (Least Concern and Near Threatened) according to the latest Finnish Red List of species^53^. We computed habitat specialization scores for each species using species habitat class information as derived from IUCN (available at www.iucn.org). We expressed diet specialization using the diet as derived from Ref.^54^. In order to derive a habitat and diet specialization index for each species, we computed the Gini coefficient (using the Gini function from R package DescTools), an index of statistical dispersion ranging from 0 to 1, reflecting a gradient from low to high specialization. This index has been recently used as a measure of habitat specialization in birds^55^. Species body mass was derived from Ref.^54^. Furthermore, we also computed an index representing the proportion of the vertebrates (both endotherm and ectotherm) in the species diet (derived from Ref.^54^, used here as a proxy for the species trophic level.

To test for phylogenetic structuring in the model residuals (see below), we derived a phylogenetic tree for our bird and mammal species from www.vertlife.org^56^.

### Matching protected and unprotected sites

To reach a balanced study design that would minimize the confounding effects stemming from landscape differences in protected and unprotected sites, we employed a matching approach. We matched each protected survey route with the most similar unprotected route based on the seven environmental variables as detailed above. These variables are deemed broadly relevant for most of the species considered^57,58^. Furthermore, we imposed a matching of sites within the same vegetation zone across Finland (overall three zones: Hemi and South Boreal pooled together, Middle Boreal, North Boreal, as shown in Fig. 6) to ensure spatial matching, e.g. protected sites could only be matched to unprotected sites within the same vegetation zone. Similarly, we also imposed matching of sites within the same Corine land cover period, so as to ensure that protected sites in e.g. period 1999–2001 are only matched with unprotected sites in the same period. For the birds only, given the large variance in survey effort in the protected area data as compared to unprotected, we also included the effort (overall km length of line transect) as an additional matching criterion. This is aimed to balance the data by matching protected and unprotected sites based on similar effort, in addition to the other criteria above, thus allowing reliable comparisons. For the matching, we chose a one-to-one nearest-neighbor covariate matching without replacement and using a caliper value of 0.25 standard deviation of the propensity scores, and tested the performance of two commonly applied matching methods, the Mahalanobis distance metric and the propensity score matching (see e.g. Refs.^18,27,28^). An assessment of the performance level for the matching done with either method suggested that the propensity score matching gives better results, that is, it can identify more similar pairs of protected and unprotected sites across most of the criteria considered as compared to the Mahalanobis distance method (see Support Figs. S2, S3) for both birds and mammals. This matching based on propensity score allowed to match most of the protected sites in the initial data (70.2% out of 510 and 91.2% out of 1553 sites by period for birds and mammals respectively) with a comparable and very similar unprotected site.

### Calculating species ecological niche

We characterized the multidimensional niche of each species (*n* = 96 birds and 22 mammals) using *n*-dimensional hypervolumes^19,59^. For each species, we constructed a hypervolume for each protection status (protected or unprotected) and the four sampling periods (note that we pooled all occurrences for the three survey years in each period, e.g., all observations from the year 1999, 2000 and 2001). This was done because sites within a period share the same environmental covariates across the three years in that period. We generated each *n*-dimensional hypervolumes using the *hypervolume* R function^19^, selecting the seven environmental axes described above, a gaussian kernel density estimator, and an optimal bandwidth for each axis as estimated via the *estimate_bandwidth* R function^19^.

We used the R package *BAT*^60^ to calculate niche metrics for each *n*-dimensional hypervolume. As a measure of niche size, we used the total volume of the *n*-dimensional hypervolume. For each species, we assessed niche differentiation between protected and unprotected areas within each sampling period. In particular, we measured niche overlap with the *kernel.beta* R function^20^ that expressed total niche differentiation as the sum of two components: niche shifts (i.e. replacement of space between hypervolumes) and niche expansion (i.e. net difference between hypervolumes^22^). Total niche differentiation (shift + expansion) varies from 0 (complete overlap between hypervolumes) to 1 (complete separation between hypervolumes). Note that, to minimize spurious estimations of niche metrics, we excluded hypervolumes where a species occurred less than five times within a given period and protection category. To ensure comparability across taxa, we excluded low dimensional hypervolumes with less than five axes. This minimizes issues of no use of a specific axis, i.e. habitat, by a given species in a given period and protection category, which would compromise the comparison of niche metrics for that specific combination.

### Statistical analyses

We tested our working hypothesis via regression-type analyses, following the protocol by Ref.^61^ for data exploration, model construction, and validation. We built generalized linear mixed effects models using the R package glmmTMB^62^, assess the relationship between species traits (independent variables) and niche expansion and niche shift within and outside protected areas (dependent variables). Prior to model fitting, we carried out data exploration by plotting variables distribution, checking for the presence of outliers, and testing the degree of collinearity among predictors^63^. As a result, we log-transformed body mass and generation length to homogenize their distribution and minimize the effect of outliers. For the same reason, we transformed the variable diet by angular transformation (i.e., arcsine square root transformation), suitable for proportional data^64^. We scaled each continuous predictor to zero mean and standard deviation one to facilitate computation and interpretation of the effect sizes. We performed scaling of the variables separately for bird and mammals, as we were not interested in making explicit comparisons between the two groups. Finally, we explored the collinearity among the candidate predictor variables with Pearson’s *r* correlations (see Figs. S6, S7), removing generation length from the model of both mammals and birds being it collinear with body mass (both *r* > 0.7)^65^.

We ran four separate models, one for each of the two dependent variables, that is niche expansion and niche shift, and separately for birds and mammals. The number of observations (after filtering as detailed in the previous section) for birds was 276 from 93 species, and for mammals 74 observations from 21 species. As the response variables vary continuously between 0 and 1, we used a beta error distribution and a logit link function. Predictor variables included the red list status as a categorical variable (levels: threatened vs not threatened), and habitat specialization, diet specialization, vertebrate proportion in the species diet (hereafter named carnivore for simplicity), and log-transformed body mass as continuous variables. We scaled each continuous predictor to zero mean and standard deviation one to facilitate computation and interpretation of the effect sizes. We performed scaling of the variables separately for bird and mammals, as we were not interested in making explicit comparisons between the two groups. In each model, we included species and period identity as factors with random effects (on the intercept). This was to account for pseudo-replication stemming from repeated measures of the same species over different periods, and for potential unexplained variation in overall niche metrics between periods.

We carried out model validation with the function *check_model* in the R package *performance*^66^ (see Figs. S8–S11). We also assessed unexplained phylogenetic signal in the model residuals using the function *phylosig* from R package *phytools*^67^. Specifically, we tested whether residuals from closely related species in the phylogenetic tree are more similar than those of more distantly related species. If a signal in the residuals is detected, this might indicate a non-independence of the sample units across the phylogeny, in a much similar way as done for testing spatial autocorrelation in model residuals^68^.

We performed all analyses in R version 4.1.0^69^. R codes to reproduce the analyses are available in GitHub (see Data availability section).

## Supplementary Information

Supplementary Information.

## Data Availability

Data supporting the study are available from FINBIF (www.laji.fi) for the birds outside of protected areas as open data, and from LUKE (Natural Resources Institute Finland) upon request. R codes to reproduce the analyses are available in GitHub (https://github.com/StefanoMammola/Santangeli-et-al-Niche-PA.git).
