# Supplementary material for: The effects of protected areas on the ecological niches of birds and mammals
Source: Sci Rep. 2022 Jul 8;12:11601. doi: 10.1038/s41598-022-15949-2 (PMC9270413; doi:10.1038/s41598-022-15949-2)
Supplement: Supplementary file 1 — Supplementary Information. [file 41598_2022_15949_MOESM1_ESM.pdf]

The effects of protected areas on the ecological niches of birds and mammals

*Andrea Santangeli** [1], *Stefano Mammola** [2,3], *Aleksi Lehikoinen*[4], *Ari Rajasärkkä*[5], *Andreas Lindén*[6], *Marjo Saastamoinen*[1,7]

* These authors share the first authorship

Correspondence to: andrea.santangeli@helsinki.fi; tel: +358 504484443; fax: +358 2941 57694

SUPPLEMENTARY INFORMATION

Table S1. The 7 land use classes used for the study, along with their matching corine land cover classification and detailed description.

**Habitat classes used for study:**	**Corine Land Cover class match**	**description:**
Artificial surfaces	1. Artificial Surfaces, includes all lower level classes	All man made surfaces including urban infrastructures, industrial settings, including mine, dump and construction sites, as well as artificial non-agricultural vegetated areas
Agricultural land	2.1 Arable land + 2.3 Pastures + 2.4 Heterogeneous agricultural	All cultivated areas for crops and fodder, typically under some rotation regime
Broad leaved forests	3.1.1 Broad leaved forests	Forest most frequently composed of Betula spp.
Coniferous forests	3.1.2 Coniferous forests	Forest most frequently composed of Picea spp. and/or Pinus spp.
Mixed forests	3.1.3 Mixed forests	Forest most frequently composed of a mixture of coniferous tree species with broad-leaved deciduous tree species
Shrub and/or herbaceous vegetation	3.2 Shrub and/or herbaceous vegetation associations	Shrubby formation with sparse trees composed of dwarf forms of *Betula spp.* and *Salix spp.* , plus *Vaccinium spp. Empetrum migrum, Ledum palustre, Carex spp., Cladonia spp.,* etc. frequently interspersed by rock outcrops. This class also includes moors and heathland
Water	4. Wetlands + 5. Water bodies	Sea and inland wetlands, including mires and bogs

Table S2: Model results testing the relationship between species traits and ecological niche differentiation processes between protected and unprotected areas. Results are given for the four separate models on niche shift and expansion in birds, and niche shift and expansion in mammals. Statistics show the shows the estimated standardized coefficients β ± SE for the effect of each trait, as well as the confidence interval, test statistics (z) and p-value. Standard deviations for the random components of each model are also reported (two rightmost columns). All continuous variables have been scaled to zero mean and 1 SD. For the red list status, the non-threatened status is used as a reference category. The sample size was 93 for the bird and 21 for the mammal models, respectively.

**Birds - Niche shift**	**Coefficient**	**SE**	**95% CI**	**z**	**p**		
Intercept	-0,3	0,08	[-0.46, -0.13]	-3,54	< .001		
Body mass	0,14	0,08	[-0.01, 0.30]	1,83	0,067	**Random part**	
Habitat specialization	0,09	0,07	[-0.05, 0.24]	1,26	0,206	**Parameter**	**Coefficient**
Diet specialization	-0,05	0,08	[-0.16, 0.15]	-0,07	0,948	SD (Intercept: year)	0,00
Diet - vertebrates	-0,13	0,07	[-0.27, 0.02]	-1,73	0,084	SD (Intercept: species)	0,42
Red listed (Yes)	0,05	0,24	[-0.42, 0.53]	0,22	0,826	SD (Residual)	1,78
**Birds - Expansion**							
Intercept	-3,98	0,14	[-4.26, -3.71]	-28,17	< .001		
Body mass	-0,04	0,1	[-0.24, 0.15]	-0,45	0,656		
Habitat specialization	0,09	0,1	[-0.10, 0.27]	0,89	0,375		
Diet specialization	-0,02	0,1	[-0.21, 0.18]	-0,18	0,859	SD (Intercept: year)	0,15
Diet - vertebrates	0,04	0,08	[-0.13, 0.20]	0,44	0,663	SD (Intercept: species)	0,76
Red listed (Yes)	-0,02	0,32	[-0.65, 0.61]	-0,07	0,945	SD (Residual)	5,68
**Mammals - shift**							
Intercept	-0,33	0,11	[-0.55, -0.10]	-2,87	0,004		
Body mass	-0,09	0,12	[-0.32, 0.15]	-0,73	0,468		
Habitat specialization	-0,11	0,1	[-0.31, 0.10]	-1	0,316		
Diet specialization	0,04	0,1	[-0.16, 0.25]	0,4	0,687	SD (Intercept: year)	0,04
Diet - vertebrates	-0,11	0,12	[-0.35, 0.13]	-0,9	0,371	SD (Intercept: species)	0,37

Red listed (Yes)	-0,04	0,29	[-0.61, 0.52]	-0,14	0,886	SD (Residual)	4,39
**Mammals - Expansion**							
Intercept	-3,12	0,1	[-3.32, -2.92]	-30,68	< .001		
Body mass	-0,13	0,09	[-0.32, 0.06]	-1,36	0,173		
Habitat specialization	-0,02	0,09	[-0.20, 0.16]	-0,23	0,816		
Diet specialization	0,12	0,09	[-0.05, 0.30]	1,4	0,162	SD (Intercept: year)	0,00
Diet - vertebrates	0,02	0,1	[-0.17, 0.21]	0,23	0,818	SD (Intercept: species)	0,00
Red listed (Yes)	-0,48	0,27	[-1.02, 0.06]	-1,74	0,081	SD (Residual)	6,23

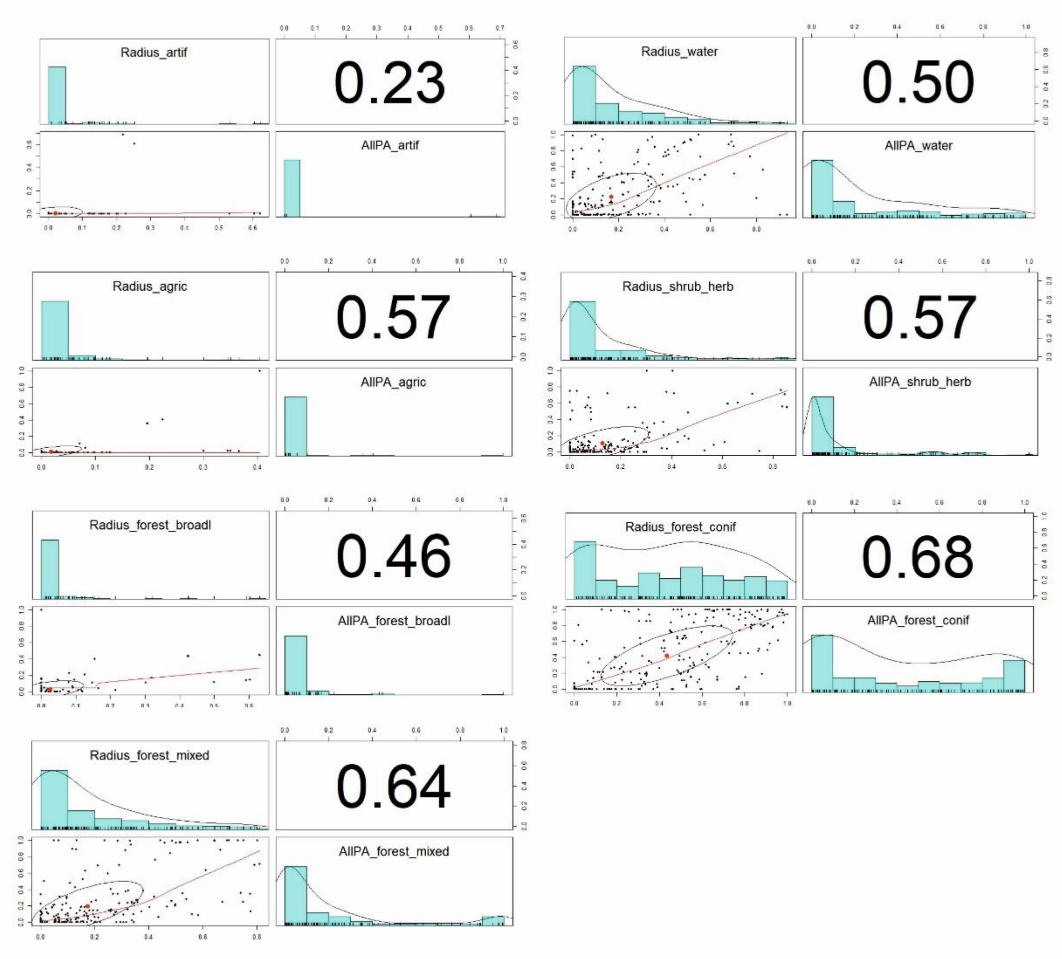

Figure S1 Correlation in the 7 environmental variables used for matching of the birds data when they are extracted using a circular radius centred at the centroid of the protected area (Radius_ variables, top left in each panel), as compared to when they are extracted across the whole area of the protected area (AllPA_ variables, bottom right in each panel). The high correlation for most of the variables between the two alternative approaches to derive the environmental variables indicates low sensitivity to this choice. For consistency with the other data on birds in unprotected land, and with the mammal data, we thus chose to use the radius based approach for extracting the environmental covariates for the protected area data for the birds.

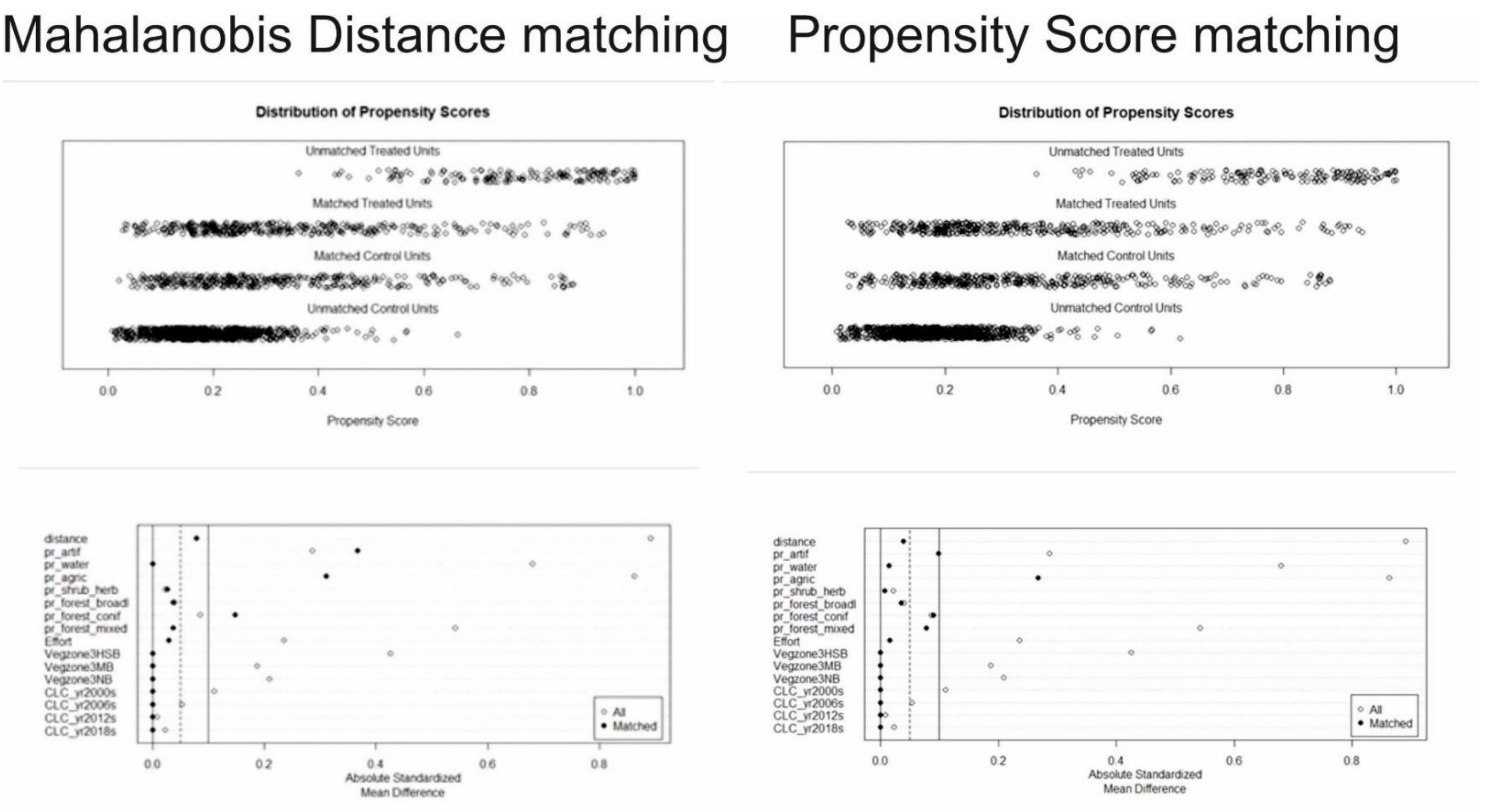

Figure S2. The impact and performance of two approaches, Mahalanobis distance (left panels) and propensity score matching (right panel) in identifying a set of similar, and thus comparable, treated (protected) and control (unprotected) sites for the bird data. Upper panels show the differences in the distribution of overall propensity scores between the treatment (protected) and control (unprotected) sites before the matching (unmatched) and after the matching (matched). Lower panels depict the absolute standardised mean difference between treatment and control sites relative to each variable used for the matching. A perfect match would have a value of zero for the difference, whereas a very poor match a value closer to one. Note the impact of the matching in reducing the mean difference between treatment and control sites as evident by the black dots (matched) shifting towards the left (reduced difference) compared to the original unmatched dataset. Differences in the matched variables relating to vegetation zone and CLC_year (Corine Land Cover periods) are equal to zero because we imposed an equal match within each vegetation zone and within each of the four CLC periods. Matched variables in the lower panels are, from the top: proportion of artificial surface, water, agricutlral land, shrub and herbaceous vegetation, broad leaved forest, coniferous forest, mixed forest, effort (the length of the line transect, see methods in the main text), the three vegetation zones and the four CLC periods. Note the better performance of the propensity score matching method compared to Mahalanobis distance (lower panels) in reducing the mean difference between treated and control sites for most of the variables.

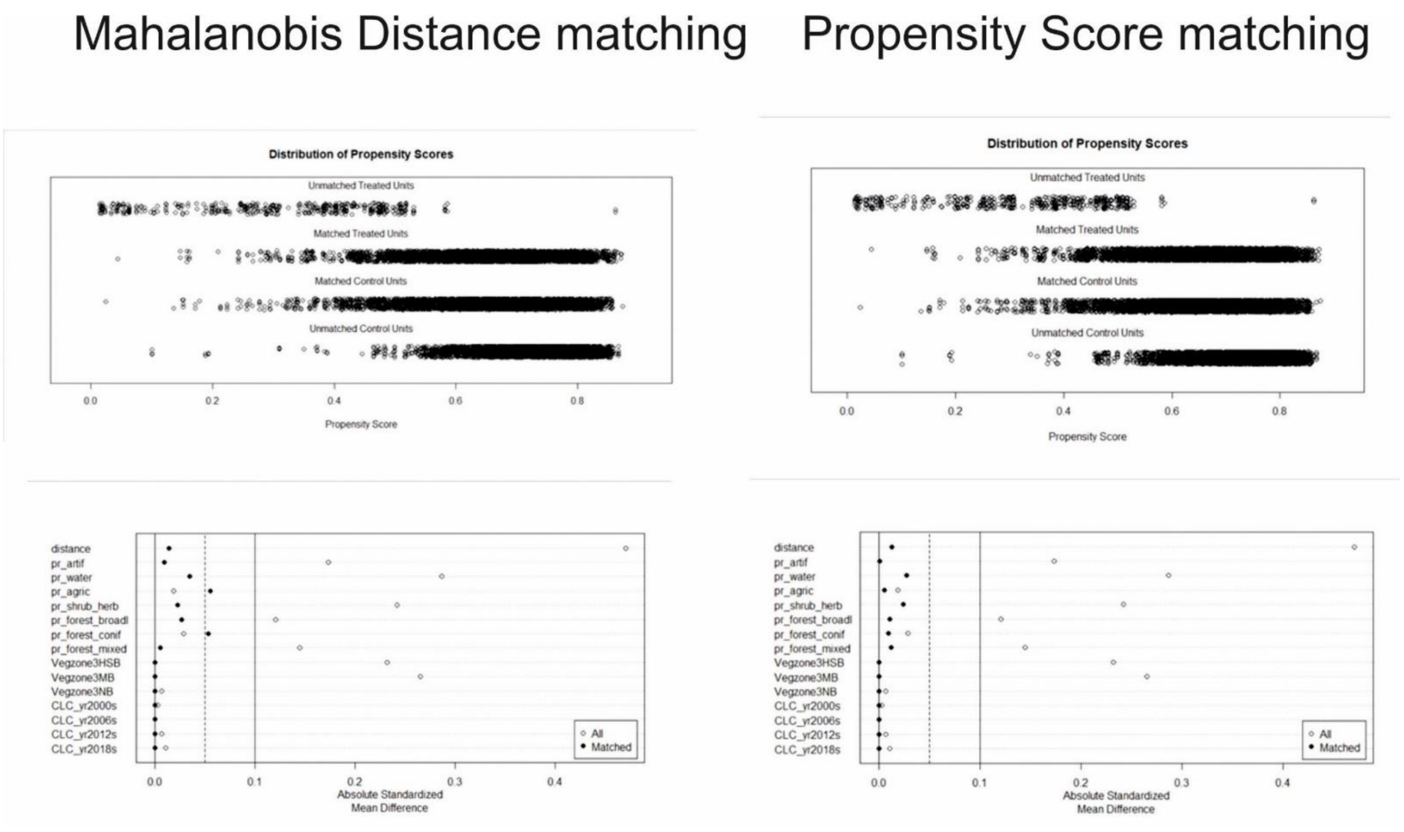

Figure S3. The impact and performance of two approaches, Mahalanobis distance (left panels) and propensity score matching (right panel) in identifying a set of similar, and thus comparable, treated (protected) and control (unprotected) sites for the mammal data. Upper panels show the differences in the distribution of overall propensity scores between the treatment (protected) and control (unprotected) sites before the matching (unmatched) and after the matching (matched). Lower panels depict the absolute standardised mean difference between treatment and control sites relative to each variable used for the matching. A perfect match would have a value of zero for the difference, whereas a very poor match a value closer to one. Note the impact of the matching in reducing the mean difference between treatment and control sites as evident by the black dots (matched) shifting towards the left (reduced difference) compared to the original unmatched dataset. Differences in the matched variables relating to vegetation zone and CLC_year (Corine Land Cover periods) are equal to zero because we imposed an equal match within each vegetation zone and within each of the four CLC periods. Matched variables in the lower panels are, from the top: proportion of artificial surface, water, agricutlral land, shrub and herbaceous vegetation, broad leaved forest, coniferous forest, mixed forest, effort (the length of the line transect, see methods in the main text), the three vegetation zones and the four CLC periods. Note the better performance of the propensity score matching method compared to Mahalanobis distance (lower panels) in reducing the mean difference between treated and control sites for most of the variables.

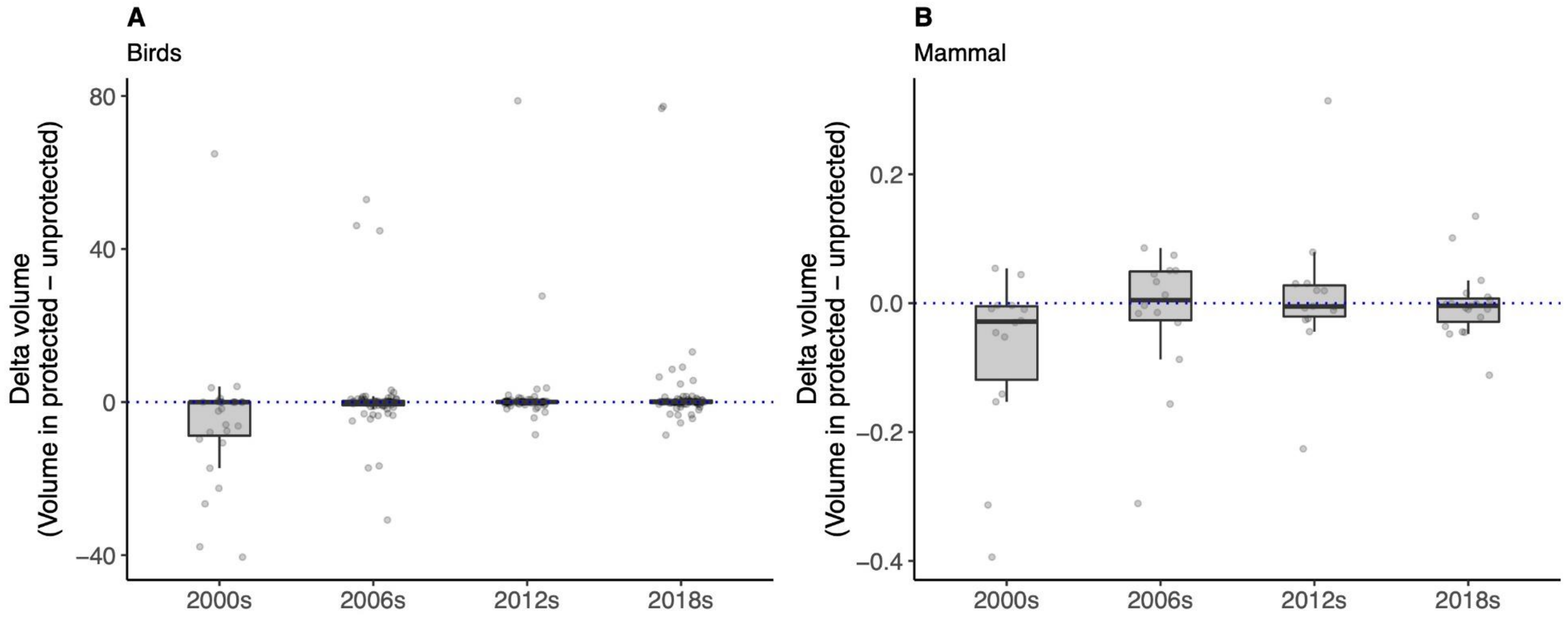

Figure S4. Difference in niche volume between protected and unprotected for (A) birds and (B) mammals. Positive values depict larger volumes in protected areas and negative values depict larger volumes in unprotected areas. Values close to zero indicate no expansion or contraction between protected and unprotected areas. Each box corresponds to each of the four periods considered for this study. Grey dots depict observed values jittered to aid visualization.

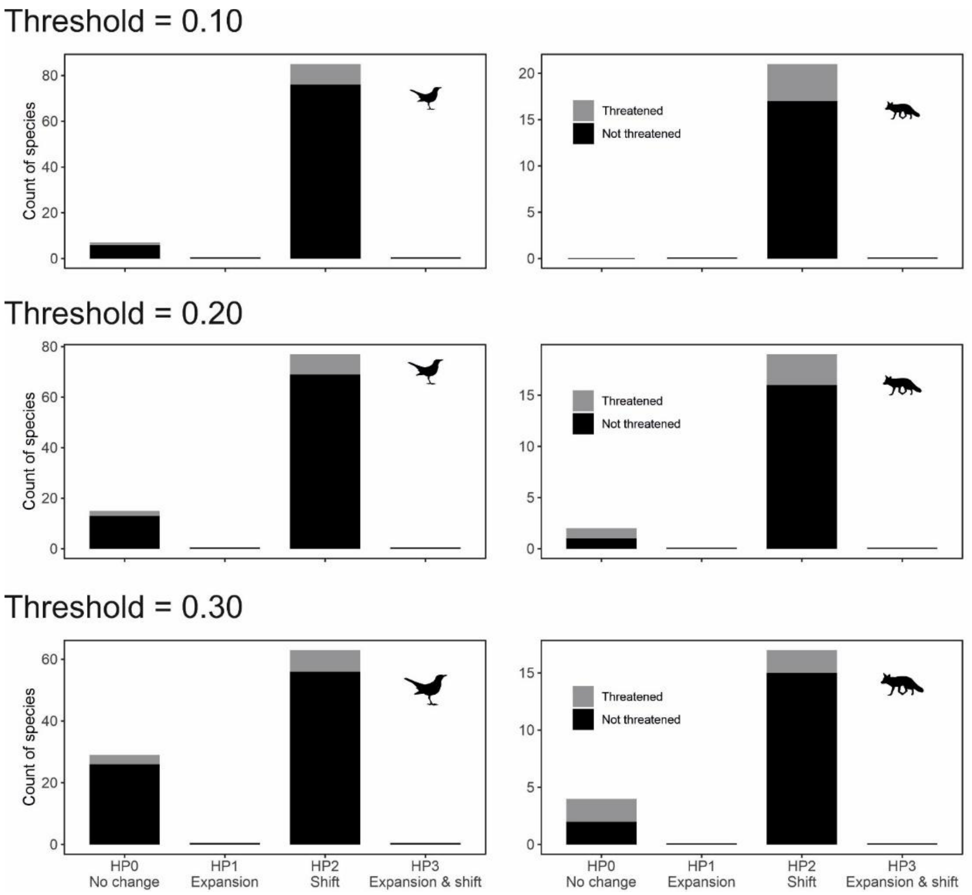

Figure S5. Bar charts showing the sensitivity of the number of species fitting each of the three hypotheses plus the null hypothesis (as presented in Figure 1 and 2 in the main text) when different thresholds (from top: 0.10, 0.20 and 0.30, respectively) are applied to separate niche change versus niche stasis. That is, the continuous differentiation in the ecological niche, either volume or habitat shift, was separated so that values below the threshold represent no change for the specific niche metric, and values above the threshold represent change. Left panels depict results for birds, and right panels for mammals. HP0 represents no expansion in the volume of the niche and no shift in habitat, HP1 represents only expansion in the volume, HP2 only shift in habitat, and HP3 represents both expansion in the volume of the niche and shift in habitat.

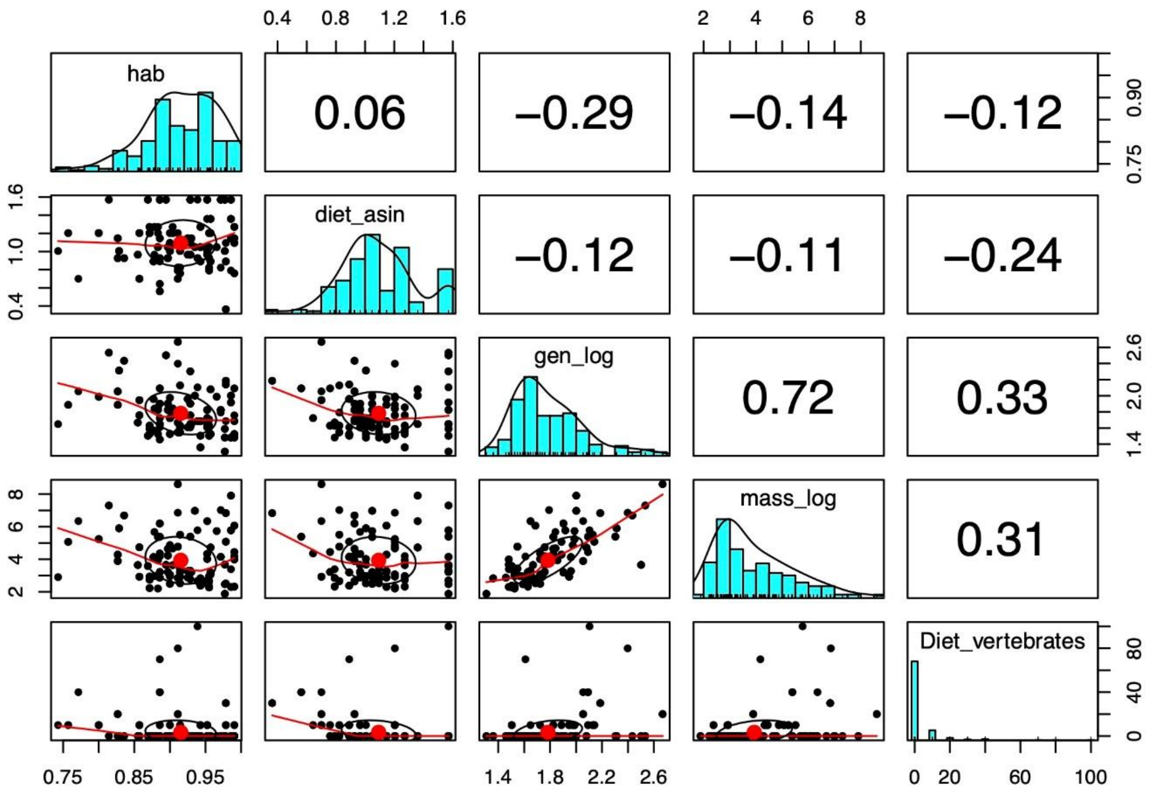

Figure S6. Collinearity plots showing the correlation between each combination of the five candidate trait predictors considered for the bird model (results presented in Fig. 5). The full variable names, along the diagonal from top left to bottom right are: Habitat specialization, Diet specialization, generation length (log transformed), body mass (log transformed), and percentage of the diet based on vertebrates (referred to as Carnivore in Figure 5). Due to the high collinearity between body mass and generation length, the latter was excluded from the trait based model.

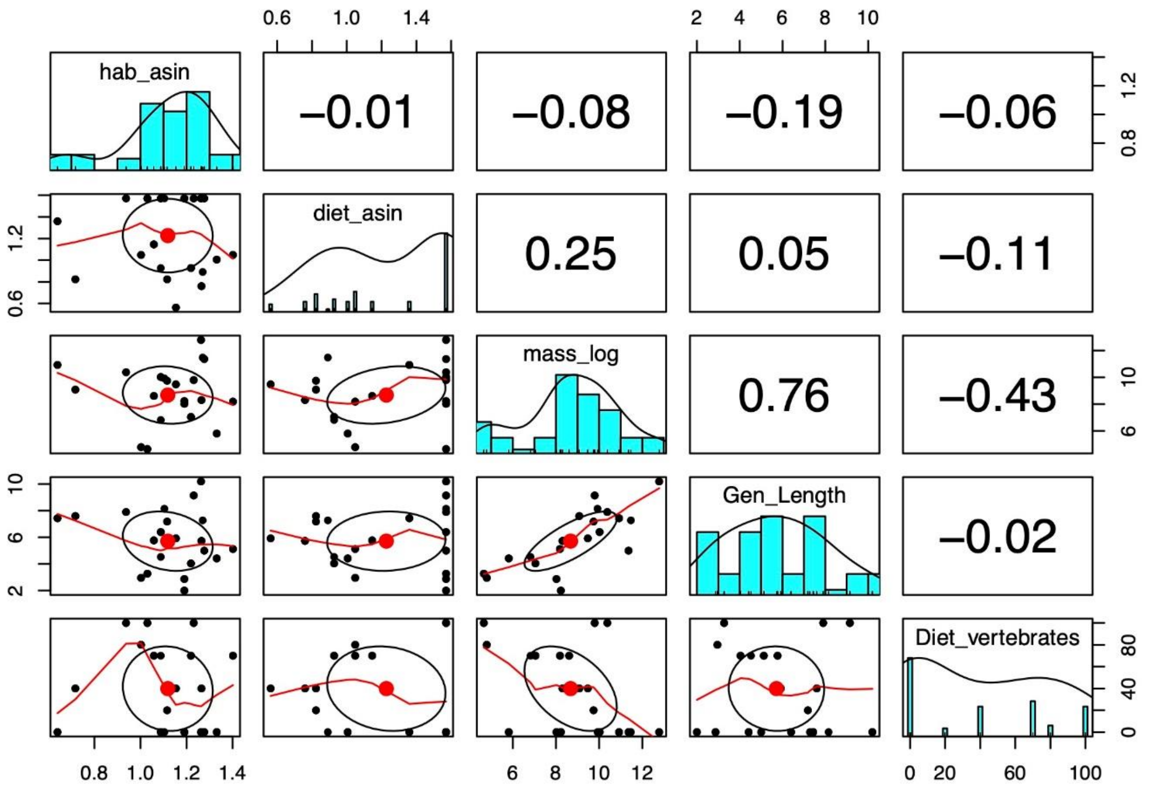

Figure S7. Collinearity plots showing the correlation between each combination of the five candidate trait predictors considered for the mammal model (results presented in Fig. 5). The full variable names, along the diagonal from top left to bottom right are: Habitat specialization, Diet specialization, generation length (log transformed), body mass (log transformed), and percentage of the diet based on vertebrates (referred to as Carnivore in Figure 5). Due to the high collinearity between body mass and generation length, the latter was excluded from the trait based model.

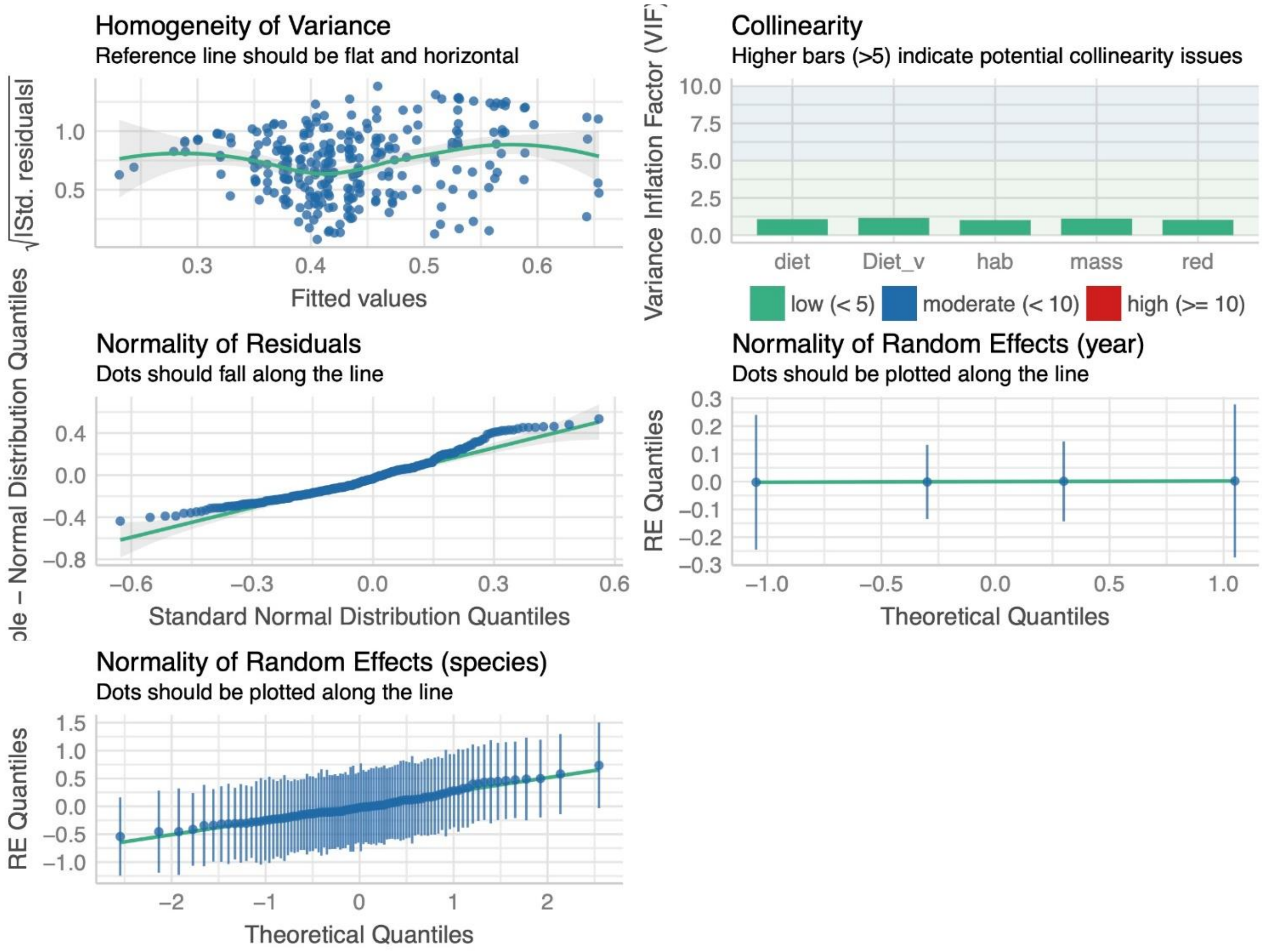

Figure S8. Performance plots relative to the bird niche expansion model (results presented in Fig. 5A).

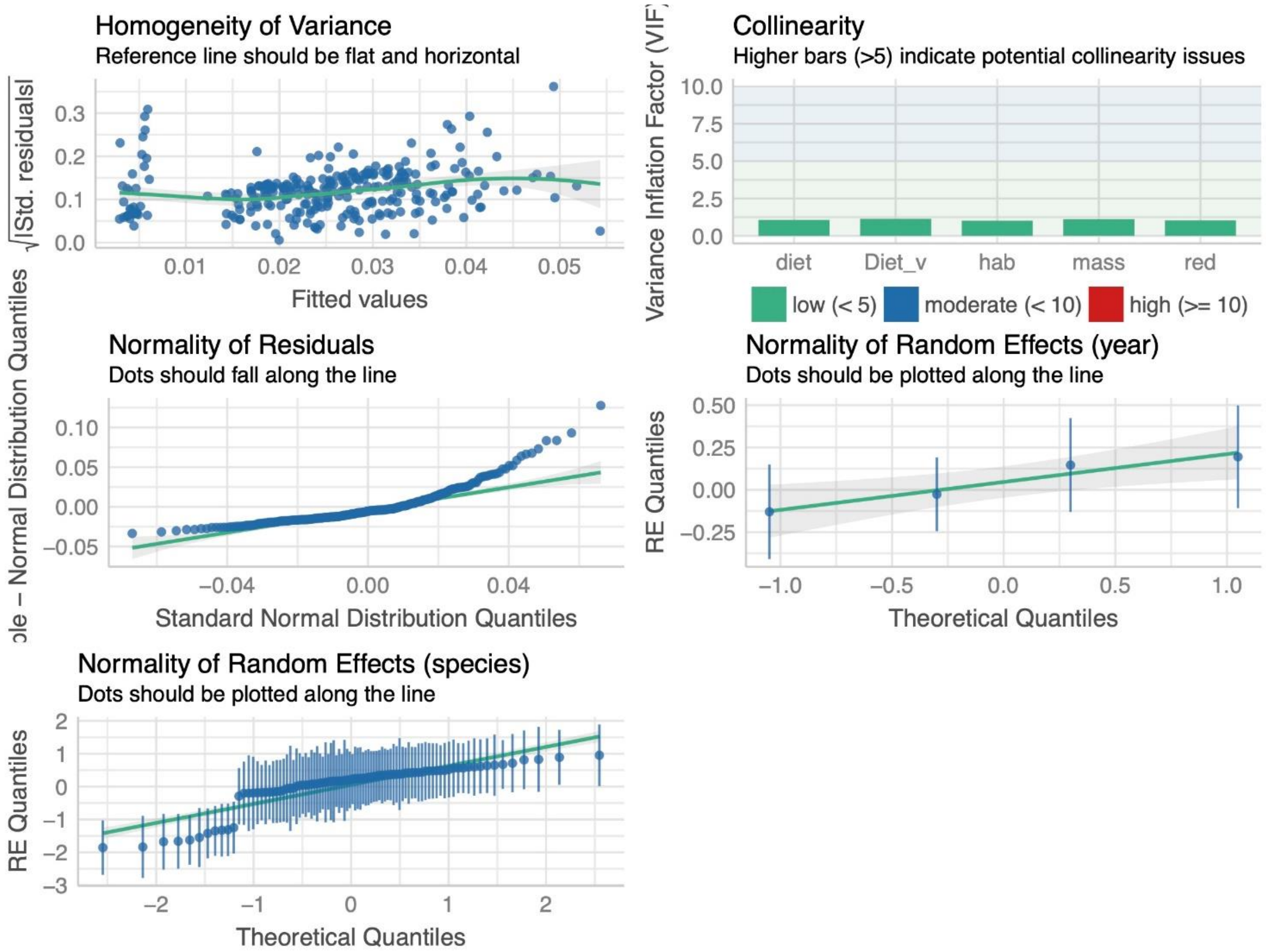

Figure S9. Performance plots relative to the bird niche shift model (results presented in Fig. 5C).

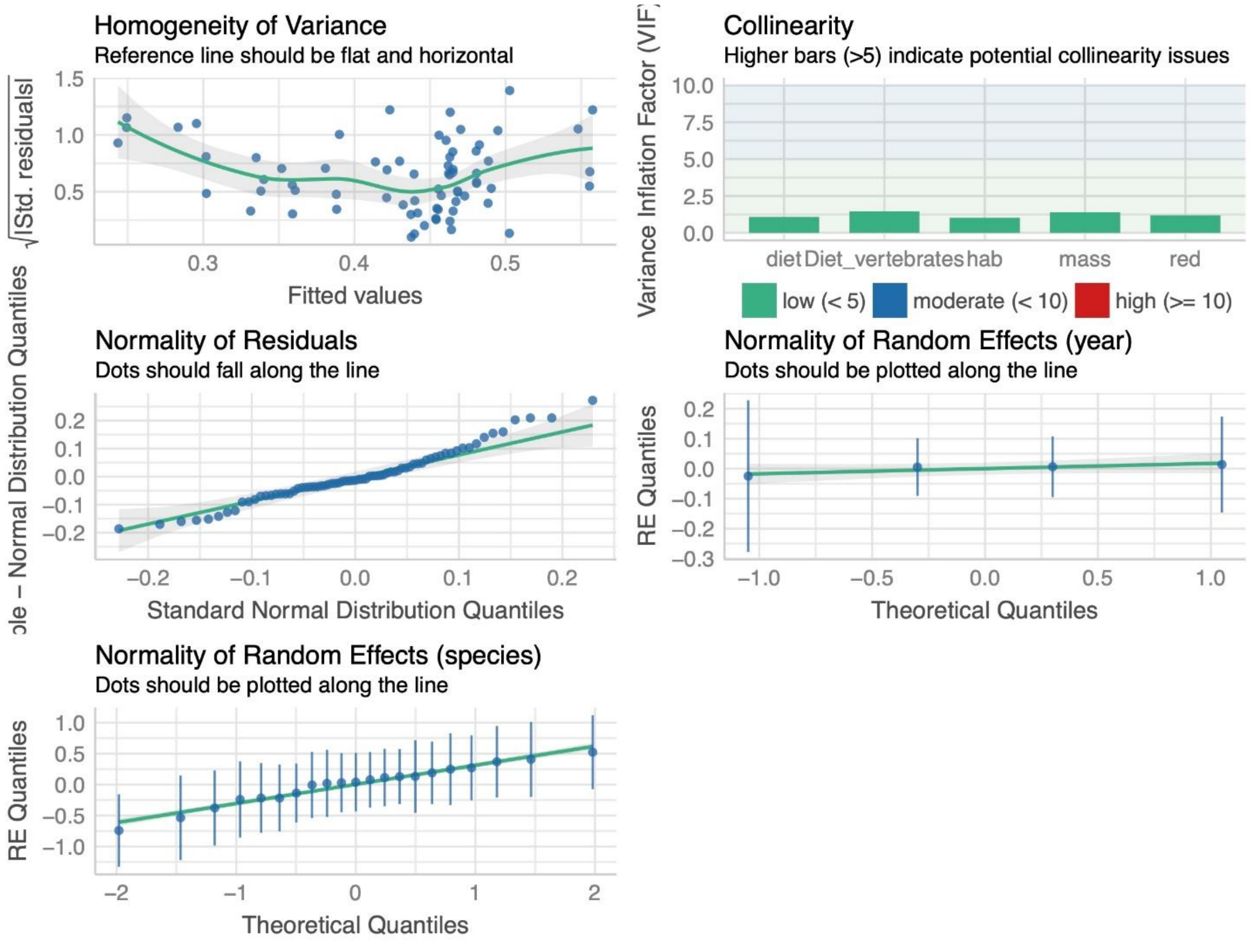

Figure S10. Performance plots relative to the mammal niche expansion model (results presented in Fig. 5B).

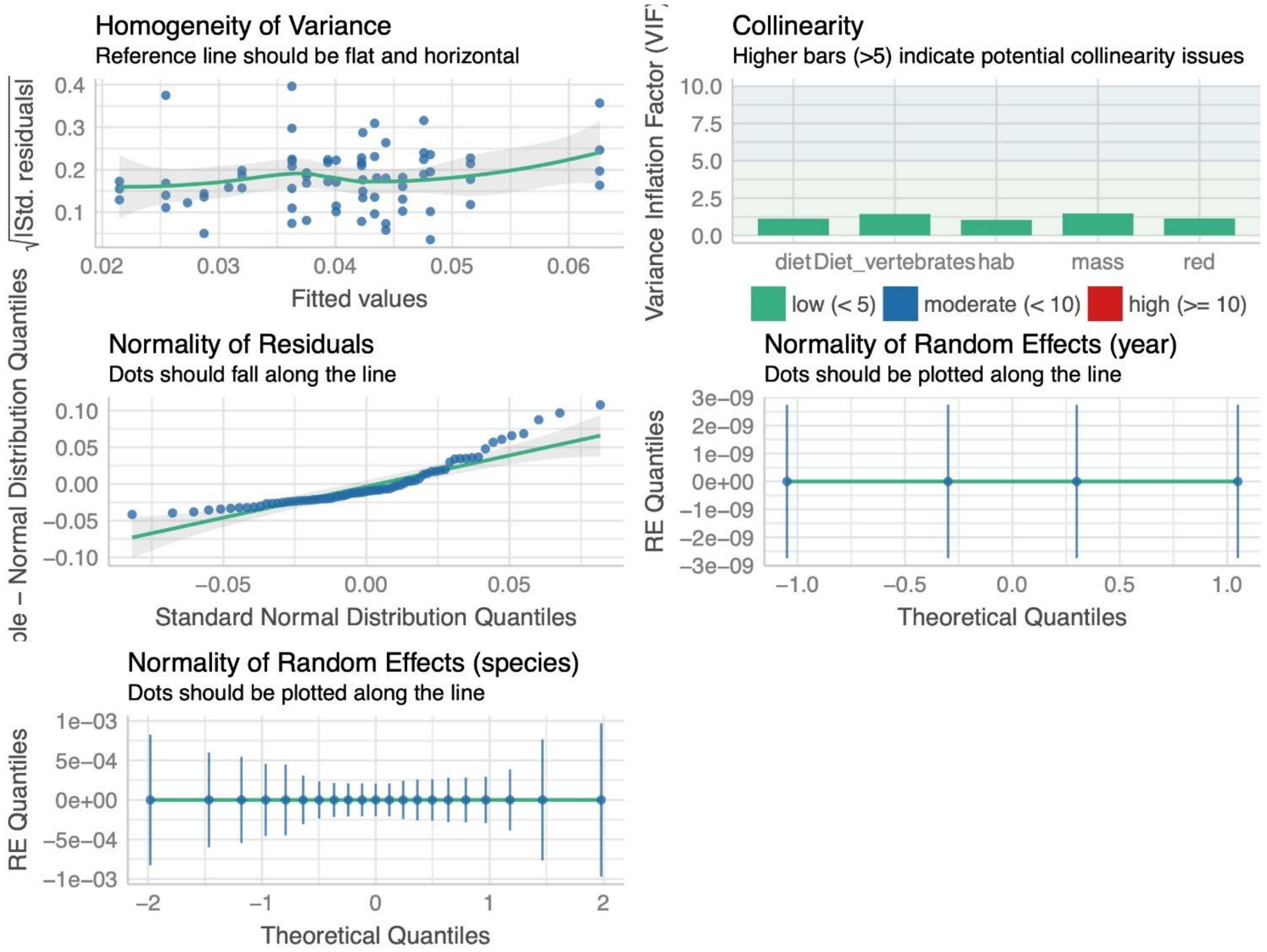

Figure S11. Performance plots relative to the mammal niche shift model (results presented in Fig. 5D).
